# Standard operating procedure changed pre-hospital critical care anaesthesiologists’ behaviour: a quality control study

**DOI:** 10.1186/1757-7241-21-84

**Published:** 2013-12-05

**Authors:** Leif Rognås, Troels Martin Hansen, Hans Kirkegaard, Else Tønnesen

**Affiliations:** 1 Department of research and development, Norwegian Air Ambulance Foundation, P.O. Pox 94, Drøbak, 1441 Norway; 2 Pre-hospital Critical Care Team, Department of Anaesthesiology, Viborg Regional Hospital, Heibergs Allé 4, Viborg, 8800 Denmark; 3 Pre-hospital Critical Care Team, Aarhus University Hospital, Oluf Palmes Allé 32, Aarhus N, 8200 Denmark; 4 Department of Pre-hospital Medical Services, Central Denmark Region, Oluf Palmes Allé 34, Aarhus N, 8200 Denmark; 5 Centre for Emergency Medicine Research, Aarhus University Hospital, Trøjborgvej 72-74, Building 30, Aarhus N, 8200 Denmark; 6 Department of Anaesthesiology, Aarhus University Hospital, Nørrebrogade 44, Aarhus, 8000 Denmark

**Keywords:** Pre-hospital, Out-of-hospital, Prehospital emergency care (MeSH), Emergency medical services (MeSH), Helicopter emergency medical service, Critical care (MeSH), Controlled ventilation, Standard operating procedure, Airway management (MeSH), Endotracheal intubation (MeSH), Patient safety

## Abstract

**Introduction:**

The ability of standard operating procedures to improve pre-hospital critical care by changing pre-hospital physician behaviour is uncertain. We report data from a prospective quality control study of the effect on pre-hospital critical care anaesthesiologists’ behaviour of implementing a standard operating procedure for pre-hospital controlled ventilation.

**Materials and methods:**

Anaesthesiologists from eight pre-hospital critical care teams in the Central Denmark Region prospectively registered pre-hospital advanced airway-management data according to the Utstein-style template. We collected pre-intervention data from February 1^st^ 2011 to January 31^st^ 2012, implemented the standard operating procedure on February 1^st^ 2012 and collected post intervention data from February 1^st^ 2012 until October 31^st^ 2012. We included transported patients of all ages in need of controlled ventilation treated with pre-hospital endotracheal intubation or the insertion of a supraglottic airways device. The objective was to evaluate whether the development and implementation of a standard operating procedure for controlled ventilation during transport could change pre-hospital critical care anaesthesiologists’ behaviour and thereby increase the use of automated ventilators in these patients.

**Results:**

The implementation of a standard operating procedure increased the overall prevalence of automated ventilator use in transported patients in need of controlled ventilation from 0.40 (0.34-0.47) to 0.74 (0.69-0.80) with a prevalence ratio of 1.85 (1.57-2.19) (p = 0.00). The prevalence of automated ventilator use in transported traumatic brain injury patients in need of controlled ventilation increased from 0.44 (0.26-0.62) to 0.85 (0.62-0.97) with a prevalence ratio of 1.94 (1.26-3.0) (p = 0.0039). The prevalence of automated ventilator use in patients transported after return of spontaneous circulation following pre-hospital cardiac arrest increased from 0.39 (0.26-0.48) to 0.69 (0.58-0.78) with a prevalence ratio of 1.79 (1.36-2.35) (p = 0.00).

**Conclusion:**

We have shown that the implementation of a standard operating procedure for pre-hospital controlled ventilation can significantly change pre-hospital critical care anaesthesiologists’ behaviour.

## Background

Standard Operating Procedures (SOPs) are detailed written instructions developed to achieve uniformity in the performance of a specific task. SOPs are an integrated part of many high-risk organisations e.g. aviation and the nuclear industry. Pre-hospital critical care teams, emergency medical services (EMS) and helicopter emergency medical services (HEMS) are other examples of such organisations. Several authors have reported performance data from physician-staffed pre-hospital critical care systems describing how they use SOPs in pre-hospital advanced airway management (PHAAM) [1-3] and other aspects of pre-hospital critical care [4]. The ability of SOPs to improve physician-provided pre-hospital care is however still uncertain. Bosse et al. showed that implementing an SOP for the pre-hospital treatment of severe exacerbation in chronic obstructive pulmonary disease in a physician-staffed EMS in Berlin did not improve overall guideline adherence [5]. Francis et al demonstrated that implementing an SOP for the pre-hospital treatment of acute coronary syndrome in the same physician-staffed EMS in Berlin improved some aspects of patient care, whereas other aspects were not affected [6]. The same research group also found that introducing an SOP for patient documentation did improve the quality of the patient care reports [7]. Martinon et al. from the physician-staffed *Service d’Aide Médicale Urgente (SAMU)* in Paris reported that the implementation of an SOP for pre-hospital rapid sequence intubation (RSI) and post-RSI treatment of children with severe traumatic brain injury (TBI) significantly improved quality of care on several, but not all quality indicators [8]. Hejselbaek et al. from the anaesthesiologist-staffed EMS in Copenhagen reported difficulties in getting pre-hospital critical care anaesthesiologists to follow clinical guidelines for the pre-hospital use of hypertonic saline [9]. The authors suggest that a possible solution to this may be the development and implementation of additional instructions and an intensified educational effort.

PHAAM and pre-hospital ventilatory controlled ventilation are core parts of pre-hospital critical care. There are only limited data available addressing how controlled ventilation should be applied in the pre-hospital setting, but recent guidelines address the need for controlled oxygenation and ventilation in TBI patients [10] and patients with cardiac arrest (CA) [11,12]. Hyperventilation worsens outcome in TBI patients because of decreased cerebral blood flow (CBF) [10,13,14] and EMS–induced hyperventilation and hypocapnia are well-known complications following pre-hospital endotracheal intubation (PHETI) [13,14]. Hypoventilation will cause hypercapnia which is known to result in increased intracerebral pressure (ICP) and decreased CBF in TBI patients [10]. Iatrogenic hypoventilation and hypercapnia is correlated to worsened outcome in TBI patients [13-15]. Hyperventilation may be harmful to the post-return of spontaneous circulation (ROSC) brain [11] and current guidelines expresses concern that hyperventilation in these patients may increase intrathoracic pressure thereby reducing the patient’s cardiac preload, cardiac output, and arterial blood pressure. This may subsequently result in decreased cerebral perfusion pressure and CBF [11]. Hypoventilation may also cause increased ICP and worsen metabolic acidosis in the post-ROSC patient [10]. Ventilation by a self-inflating bag may result in large tidal volume variations [16]. This could increase both the risk of hypo- and hyperventilation and the risk of high airway pressures, which in turns result in an increased risk of lung injury such as *Acute Respiratory Distress Syndrome* (ARDS) [17,18].

We postulate that providing pre-hospital controlled ventilation via an automated ventilator may increase the likelihood of achieving more optimal and stable levels of ETCO_2_. Providing controlled ventilation with optimal frequency and tidal volumes while using a self-inflating bag may be possible, but we claim that it will take most of the pre-hospital care provider’s attention span. We believe that the only realistic way to achieve these goals under stressful pre-hospital conditions while performing several other vital tasks is by ventilating the patients with an automated ventilator and adjusting its setting according to continuous measurements of ETCO_2_, SpO_2_ and peak airway pressures.

In order to ensure the use of an automated ventilator whenever feasible we therefore designed an SOP for pre-hospital controlled ventilation in the pre-hospital critical care teams in our region.

### Objective

The objective of this study was to evaluate whether the development and implementation of an SOP for controlled ventilation during transport could change pre-hospital critical care anaesthesiologists’ behaviour and thereby increase the use of automated ventilators during transport of patients ventilated via an endotracheal tube or a supraglottic airway device (SAD).

We hypothesised, that the implementation of such an SOP could significantly increase the prevalence of patients ventilated by the use of automated ventilator during transport.

## Materials and methods

### Design

This is a before-and-after quality insurance study of the implementation of an SOP in anaesthesiologist-staffed pre-hospital critical care teams.

### Setting

The data collection for this study was part of a larger prospective cohort study of pre-hospital advanced airway management in the Central Denmark Region [19,20].

The Central Denmark Region covers a mixed urban and rural area of approximately 13,000 km^2^ with a population of 1.270.000, and an overall population density is 97.7 inhabitants pr. km^2^.

The emergency medical Service (EMS) is a two-tiered system based on 64 road ambulances staffed by emergency medical technicians (EMTs) supported by ten pre-hospital critical care teams staffed with an anaesthesiologist and a specially trained EMT. Rapid response vehicles deploy nine of the pre-hospital critical care teams; the tenth team staffs a HEMS helicopter. All units are equipped with waveform capnography and an automated ventilator [19-21].

### Participants

Inclusion criteria were consecutive transported patients of all ages treated with pre-hospital endotracheal intubation or insertion of an SAD.

Exclusion criteria were inter-hospital transfers.

### Interventions

We carried out the intervention in November and December 2011 and January 2012.

The intervention consisted of:

a) Development of an evidence-based SOP for the controlled ventilation of transported patients treated with pre-hospital endotracheal intubation or insertion of an SAD by the pre-hospital critical care teams. The development of the SOP involved the clinical leads of the different pre-hospital critical care teams. We also invited the pre-hospital critical care anaesthesiologists to give feedback regarding the structure and contents of a preliminary version of the SOP.

b) Introduction of the SOP. The clinical leads and members of the research group introduced the SOP to the pre-hospital critical care teams by e-mails, lectures and group discussions. We also made the SOP available on the regional on-line collection of medical guidelines, instructions and SOPs.

The key points of the SOP were:

• Advanced airway management should be provided according to local, national or international standards.

• The attending pre-hospital anaesthesiologist decided whether or not to perform advanced airway management.

• In patients in need of controlled ventilation and treated with an endotracheal tube, a supraglottic airway device or a surgical airway, controlled ventilation should be provided by using the automated ventilator under the guidance of continuous ETCO_2_ monitoring.

• Short transport distance to the receiving hospital were not by itself considered a valid reason for not using the automated ventilator.

A translated version of the full SOP is available as Additional file 1.

c) Implementation of the SOP: We implemented the SOP on February 1^st^ 2012.

We collected post-intervention data from February 1^st^ 2012 to November 1^st^ 2012.

### Control group

From February 1^st^ 2011 to January 31^st^ 2012 we collected pre-hospital advanced airway management data from pre-hospital critical care teams according to the international consensus template [22]. Patients who during these 12 months met the inclusion criteria were the control group for the current study.

### Endpoints and variables

The primary endpoints were

1. the overall percentage of included patients ventilated on an automated ventilator

2. the percentage of included TBI patients ventilated on an automated ventilator

3. the percentage of included post-ROSC patients ventilated on an automated ventilator.

We collected all core data proposed in the consensus-based template by Sollid et al. [22] and the variables were defined as in this template. Of special interest are the following definitions:

The pre-hospital critical care physician registered the patient category. The alternatives were:

a) isolated traumatic brain injury, b) polytrauma, c) strangulation/suffocation, d) burns, e) other blunt trauma, f) penetrating trauma, g) cardiac arrest, h) cardiac disease (excluding cardiac arrest), i) asthma/chronic obstructive pulmonary disease (COPD), j) stroke/subarachnoid hemorrhage, k) ear-nose-throat (ENT) disease, l) other.

We also required the physicians to register how they ventilated the patients after performing PHAAM. The options were: 1) Spontaneous ventilation, 2) Controlled ventilation by a self-expanding bag, 3) Controlled ventilation by the automated ventilator, 4) a combination of ventilation by the self-expanding bag and ventilation by the automated ventilator, 5) a combination of controlled and spontaneous ventilation. Since the automated ventilators could only provide controlled ventilation, only patients marked as alternatives 3 and 4 (and not alternative 5) were considered as having being ventilated by the automated ventilator.

### Data sources and data collection

We collected data from eight of the ten pre-hospital critical care teams, including the HEMS. Due to differences in organisation, staffing, case mix and caseload, the last two teams were not part of the study. The anaesthesiologists in the participating teams filled in a registration form containing all the core data recommended by Sollid et al. [22] as well as the specific variables listed above. A translated version of the registration form is available as Additional file 2. We have described data collection and handling in more detail elsewhere [19].

### Bias

To reduce the risk of recall bias and selection bias, the primary investigator reviewed the registration forms on a day-to-day basis. We crosschecked the registration forms with the standard pre-hospital records from the pre-hospital critical care teams to ensure the highest possible data coverage. In cases of missing data or inconsistencies, we asked the attending pre-hospital critical care anaesthesiologist to provide additional details for clarification.

### Study size

We expected, based on experience from the system involved, that the prevalence of automated ventilator use in patients in need of controlled ventilation before the introduction of the SOP would be approximately 30% and that the SOP could increase this prevalence to 60%. Sample size calculations made in the statistical program *Stata 12 (StataCorpLP)* showed that it would require 63 patients in each group to detect a difference of this magnitude with 90% power at a significance level of 5%.

### Statistical methods

We analysed the data in *Stata 12 (StataCorpLP)* and tested the hypotheses of no association using the *chi-squared test* except when data were scarce, in which case we applied *Fisher’s exact test*. We give estimates with 95% confidence intervals (CI) and consider a p-value below 0.05 as statistically significant. Because of the rigorous crosschecking and day-to-day control, missing data were rare. If we could not obtain the missing data, we performed complete case analyses.

### Ethics

This was a quality control study, testing whether the SOP could improve the quality of patient care. The study did not involve any alterations from normal practice and according to Danish law, it did not need the approval of the Regional Ethics Committee.

The Danish Data Protection Agency approved the study (Journal number 2013-41-1462).

## Results

We included 515 patients. In total, six transported patients had an SAD inserted as an airway back-up device. The rest of the included patients (n = 509) had their tracheas intubated. The attending anaesthesiologists ventilated all the patients who had an SAD inserted by using a self-expanding ventilation bag.

In Table 1, we display the results of implementing the SOP on the overall prevalence of automated ventilator use during transport of patients treated with PHETI or an SAD.

**Table 1:** The effect of introducing an SOP^* ^**for pre-hospital controlled ventilation on the use of automatic ventilators**

**Ventilator used**	**Before SOP (CI)**	**After SOP (CI)**	**Total**
**Yes**	100	198	298
**No**	149	68	217
**Total**	**249**	**266**	**515**
**Prevalence**	**0.40 (0.34-0.47)**	**0.74 (0.69-0.80)**	**0.58**

*Standard operating procedure.

The SOP increased the overall prevalence of automated ventilator use from 0.40 (0.34-0.47) to 0.74 (0.69-0.80) with a prevalence ratio of 1.85 (1.57-2.19). This difference is statistically significant (p = 0.00).

We present the impact of the introduction of the SOP on the prevalence of automated ventilator use in patients with a TBI in Table 2. The SOP increased the prevalence of automated ventilator use from 0.44 (0.26-0.62) to 0.85 (0.62-0.97) with a prevalence ratio of 1.94 (1.26-3.0). This difference is statistically significant (p = 0.0039).

**Table 2:** The effect of introducing an SOP^* ^for pre-hospital controlled ventilation on the use of automated ventilators in patients with TBI^**^

**Ventilator used**	**Before SOP**	**After SOP**	**Total**
**Yes**	14	17	31
**No**	18	3	21
**Total**	**32**	**20**	**52**
**Prevalence**	**0.44 (0.26-0.62)**	**0.85 (0.62-0.97)**	**0.60**

*Standard operating procedure.

**Traumatic brain injury.

Table 3 shows the effect of the SOP on automated ventilator use in patients with ROSC after pre-hospital CA. The SOP increased the prevalence of automated ventilator use from 0.39 (0.26-0.48) to 0.69 (0.58-0.78) with a prevalence ratio of 1.79 (1.36-2.35). This difference is statistically significant (p = 0.00).

**Table 3:** The effect of introducing an SOP^* ^for pre-hospital controlled ventilation on the use of automatic ventilators in patients with ROSC^** ^after PHCA^***^

**Ventilator used**	**Before SOP (CI)**	**After SOP (CI)**	**Total**
**Yes**	42	64	106
**No**	67	29	96
**Total**	**109**	**93**	**202**
**Prevalence**	**0.39 (0.29-0.48)**	**0.69 (0.58-0.78)**	**0.52**

*Standard operating procedure.

**Return of spontaneous circulation.

***Pre-hospital cardiac arrest.

## Discussion

Our results show that implementing an SOP in a system of anaesthesiologists-staffed pre-hospital care teams can change pre-hospital critical care anaesthesiologists’ behaviour. We confirmed our hypothesis that the introduction of the SOP could significantly increase both the overall prevalence of ventilator use and the prevalence of ventilator use in transported TBI patients and patients who had achieved ROSC after pre-hospital CA.

Our result is in agreement with those reported by Martinon et al. [8] from Paris who found that the prevalence of automated ventilator use following RSI on paediatric TBI patients rose to 88% after the implementation of their guideline. Our result compares favourably to those found by Bosse et al. [5] and Francis et al. [6] from the physician-staffed EMS in Berlin. They investigated the impact of introducing SOPs for the pre-hospital treatment of acute exacerbation in COPD [5] and acute coronary syndrome (ACS) [6] and neither of the SOPs in these studies improved overall patient care. The authors introduced the SOPs by arranging staff meetings and distributing the SOP by e-mail and in paper copies. This is not very different from how we introduced the new SOP in our system. However, there are also some potentially important differences. Most importantly, both the SOP for exacerbation in COPD and the SOP for ACS are rather complex ones. They either require the physicians to learn the flow of actions described in the SOP by heart, or to have the SOP available bedside. In contrast, the SOP implemented in our pre-hospital critical care teams contained one simple lesson: “Use the ventilator!” Both Bosse and Francis found that their SOPs improved some aspects of patients care such as the correct use of some of the appropriate medications, and this may be in accordance with our result. Secondly, the SOPs for exacerbation in COPD and for ACS carry no immediate advantage (e.g. lighter workload or fried hands) for the attending physician. On the contrary, the physicians may have seen the introduction of the SOPs as an added workload or a burden. It is well known that, among other factors *“the acceptance of a guideline depends on the relevance of its topics for resolving the problems encountered” *[23]. We speculate, that the SOP for controlled ventilation introduced in our system quickly proved an advantage, decreasing the workload during patient transportation for the pre-hospital critical care anaesthesiologists once they had become accustomed to using the ventilator more frequently. We find it likely that this contributed to the satisfactory high compliance to the SOP found in this quality control study.

Several authors have described different types of barriers that may inflict the implementation of guidelines and SOPs [24-26]. They typically classify these barriers as organisational, social and professional or equivalents hereto. Organisational barriers could be financial constrains, the availability of the guidelines or the perception of the care provider. Normal routine, the opinion of leaders and the existence of obsolete medical knowledge are examples of social barriers, and professional barriers may be found in the knowledge, attitudes, self-confidence, clinical skills and coping strategies of the health-care provider [27]. When implementing the SOP for controlled ventilation, we tried to overcome these barriers by involving both the clinical leads (social barriers) and the pre-hospital critical care anaesthesiologists (organisational, social and professional barriers) in the development of the SOP. We took care in introducing the reasons for implementing the SOP both by e-mail and by conducting staff meetings (professional barriers) and made sure that the SOP was available on several platforms (organisational barriers). Our results suggest that our implementation strategy may have overcome the most important barriers.

Still, more than 25% of patients in need of controlled ventilation during transport were ventilated by a self-expanding bag. There may be several reasons for this. The most important is probably that the type of ventilator used by the pre-hospital critical care teams deployed by rapid response vehicles is not suited for all patients. They are basic volume controlled ventilators with no support mode. They do not allow any trigging of the ventilator by the patients. Patients with some degree of ventilatory effort will therefore frequently need either to be (more heavily) sedated, to be treated with a NMBA or to have their ventilation supported by self-expanding bag ventilation. A more advanced ventilator may solve some of these situations, thereby potentially increasing the prevalence of patients mechanically ventilated. On the other hand, more advanced ventilators are often more bulky and requires more education and training, both of which are factors that might reduce their use in the pre-hospital setting.

The anaesthesiologists ventilated a small portion of the patients via an SAD used as an airway back-up device. We think that not putting them on the ventilator is a reasonable choice. We did not design this study to make comparisons between the patients ventilated via an SAD and those ventilated via an endotracheal tube.

### Limitations

This was not an outcome study or a study of the quality of patient care per se. We designed the study to investigate whether the introduction of an SOP could change the behaviour of pre-hospital critical care anaesthesiologists. Information regarding the quality of the pre-hospital ventilation provided to the patients who were ventilated by a self-expanding bag compared to the quality provided by using the automated ventilator would, of course be of great interest. This, however, is beyond the scope of this study. In our opinion, evaluating the quality of the ventilation provided based on ETCO_2_-measurements would at the very least take a capnograph that were able to store continuous ETCO_2_ –data. Only then would we be able to make meaningful comparisons taking into account the frequency and degree of ETCO_2_ variations and episodes of ETCO_2_ – values outside the target range. Furthermore, defining this target range may prove difficult, especially when taking into account the results by Warner et al. showing a large degree of discrepancy between ETCO_2_ and the CO_2_–level in arterial blood (PaCO_2_) in severely injured patients [28].

Because the attending anaesthesiologists collected the data recall-and selection bias cannot be ruled out. Due to the rigorous day-to-day data control, the high response rate and no missing data we estimate the extent of these types of biases to be limited.

### Generalisability

This was part of a larger study from one homogenous Danish system of anaesthesiologist-staffed pre-hospital critical care teams. This limits the ability to generalise the findings to other systems with a different staffing, caseload or case mix. Never the less, we believe that our results may have considerable impact on similar physician-staffed pre-hospital services because they indicate the possibility of altering physician behaviour and thereby potentially improving patient care by the introduction of a relatively simple SOP to a physician-staffed pre hospital critical care service.

### Perspectives

More research is needed into the use of SOPs in physician-provided pre-hospital critical care. Especially, the best way to design and implement more complex SOPs in these settings needs to be identified.

## Conclusion

We have shown that the introduction of an SOP for pre-hospital controlled ventilation in a system of anaesthesiologist-staffed pre-hospital critical care teams can significantly affect anaesthesiologists’ behaviour.

## Abbreviations

SOP: Standard operating procedure; PHAAM: Pre-hospital advanced airway management; EMS: Emergency medical service; HEMS: Helicopter emergency medical service; ETI: Endotracheal intubation; PHETI: Pre-hospital endotracheal intubation; SAD: Supraglottic airway device; TBI: Traumatic brain injury; CA: Cardiac arrest; ROSC: Return of spontaneous circulation; EMT: Emergency medical technicians.

## Competing interests

The authors declare that they have no competing interests.

## Authors’ contributions

LR and TMH developed the SOP. LR, TMH, HK and ET designed the study. LR performed the data collection and management. All authors contributed to data analysis and interpretation. LR drafted the manuscript. TMH, HK and ET revised the manuscript critically for important intellectual content. All authors read and approved the final version of the manuscript for publication.

## Authors’ information

LR is a research fellow at the Norwegian Air Ambulance Foundation, consultant anaesthesiologist at the Viborg Regional Hospital and a pre-hospital critical care physician in the Central Denmark Region. He currently holds the post as Programme Director for the *Scandinavian Society of Anaesthesiology and Intensive Care Medicine Advanced Educational Programme in Emergency Critical Care*.

TMH is a consultant anaesthesiologist at the Aarhus University Hospital, clinical lead of the Pre-hospital Critical Care Team in Aarhus and a pre-hospital critical care physician in the Central Denmark Region.

HK is a consultant anaesthesiologist and professor of emergency medicine at the Aarhus University Hospital.

ET is a consultant anaesthesiologist and professor of anaesthesiology at the Aarhus University Hospital.

## Supplementary Material

Additional file 1 Standard Operating Procedure (SOP) controlled ventilation. Click here for file

Additional file 2 Pre-hospital Advanced Airway Management in the Central Denmark Region. Click here for file
