# Supplementary material for: Standard operating procedure changed pre-hospital critical care anaesthesiologists’ behaviour: a quality control study
Source: Scand J Trauma Resusc Emerg Med. 2013 Dec 5;21:84. doi: 10.1186/1757-7241-21-84 (PMC4029444; doi:10.1186/1757-7241-21-84)
Supplement: Additional file 1 — Standard Operating Procedure (SOP) controlled ventilation. [file 1757-7241-21-84-S1.docx]

**Pre-hospital Standard Operating Procedure (SOP)**

**Pre-hospital controlled ventilation**

**Setting:**

The pre-hospital critical care teams in the Central Denmark Region

**Objective:**

To ensure a standardised appropriate and evidence based pre-hospital treatment of patients in need of controlled ventilation.

**Patient group:**

This SOP targets all pre-hospital patients (not including neonates and infants) treated with controlled ventilation via an Endotracheal Tube (ET), a Supraglottic Airway Device (SAD) or a surgical airway by the physician-staffed Pre-hospital Critical Care Teams in the Central Denmark Region.

**Definitions:**

**Manual ventilation:**

Ventilation performed by using a self-expanding bag.

**Controlled ventilation:**

Ventilation controlled by the pre-hospital care provider by either manual ventilation or an automated ventilator.

**Automated ventilator:**

All pre-hospital critical care teams carry a mobile automated ventilator. The ventilators in the rapid response vehicles are only capable of providing volume controlled ventilation. Ventillatory support is not possible. Positive end expiratory pressure can only be provided by an external valve and the oxygen fraction in the inspired air can only be either 0.60 or 1.0. The HEMS carries a more advanced ventilator, this is described elsewhere.

**End-tidal CO2:**

End-tidal CO_2_ is defined as the measurement of CO2 content in the expired air at the end of each expiration. All pre-hospital critical care teams carry a small portable waveform capnography in the primary bag. The LP12 monitor can also be used as a waveform capnograph.

**Advanced airway management**:

This is defined as airway management beyond opening of the airway and the use of an oro-pharyngeal airway.

Possible modes of action include the use of:

- Naso-pharyngeal airway
- Standard laryngeal mask airway(LMA)
- Intubating laryngeal mask (ILMA)
- Endotracheal tube
- Surgical airway

**Standard:**

**How to provide controlled ventilation ion the pre-hospital setting:**

- Advanced airway management should be provided according to local, national or international standards.
- The decision whether or not to perform advanced airway management rests on the attending pre-hospital anaesthesiologist.
- If a patient in need of controlled ventilation is provided with an endotracheal tube, a supraglottic airway device or a surgical airway, then controlled ventilation should be provided by using the automated ventilator under the guidance of continuous ETCO_2_ monitoring.
- Short transport distance to the receiving hospital is not by itself considered valid reason for not using the automated ventilator.

**Documentation:**

This is carried out according to usual regional standards.

**Responsibilities:**

The clinical leads of the pre-hospital critical care teams are responsible for implementing this SOP in their own team. All pre-hospital anaesthesiologists are responsible for reading and applying this SOP.
